# A Cross-Domain Benchmark of Intrinsic and Post Hoc Explainability for 3D Deep Learning Models

**DOI:** 10.3390/jimaging12020063

**Published:** 2026-01-30

**Authors:** Asmita Chakraborty, Gizem Karagoz, Nirvana Meratnia

**Affiliations:** Department of Mathematics and Computer Science, Eindhoven University of Technology, 5612 AZ Eindhoven, The Netherlands; a.chakraborty@student.tue.nl (A.C.); n.meratnia@tue.nl (N.M.)

**Keywords:** explainable artificial intelligence (XAI), 3D deep learning, benchmarking framework, intrinsic explainability, post hoc explainability, quantitative evaluation, 3D medical imaging, voxelized data, 3D point cloud analysis

## Abstract

Deep learning models for three-dimensional (3D) data are increasingly used in domains such as medical imaging, object recognition, and robotics. At the same time, the use of AI in these domains is increasing, while, due to their black-box nature, the need for explainability has grown significantly. However, the lack of standardized and quantitative benchmarks for explainable artificial intelligence (XAI) in 3D data limits the reliable comparison of explanation quality. In this paper, we present a unified benchmarking framework to evaluate both intrinsic and post hoc XAI methods across three representative 3D datasets: volumetric CT scans (MosMed), voxelized CAD models (ModelNet40), and real-world point clouds (ScanObjectNN). The evaluated methods include Grad-CAM, Integrated Gradients, Saliency, Occlusion, and the intrinsic ResAttNet-3D model. We quantitatively assess explanations using the Correctness (AOPC), Completeness (AUPC), and Compactness metrics, consistently applied across all datasets. Our results show that explanation quality significantly varies across methods and domains, demonstrating that Grad-CAM and intrinsic attention performed best on medical CT scans, while gradient-based methods excelled on voxelized and point-based data. Statistical tests (Kruskal–Wallis and Mann–Whitney U) confirmed significant performance differences between methods. No single approach achieved superior results across all domains, highlighting the importance of multi-metric evaluation. This work provides a reproducible framework for standardized assessment of 3D explainability and comparative insights to guide future XAI method selection.

## 1. Introduction

Deep neural networks (DNNs) have demonstrated strong performance in learning three-dimensional (3D) data across various high-impact domains, including medical imaging [1], object recognition [2], and autonomous systems [3]. However, their lack of explainability—often described as the “black-box” problem—raises concerns about their transparency and trustworthiness, particularly in high-stake applications [4]. These concerns are amplified in 3D settings, where the spatial complexity of the input data further hinders the model’s performance [5]. Explainable Artificial Intelligence (XAI) aims to address these concerns by making AI systems’ decision-making processes more transparent and interpretable [6].

Despite growing interest in explainable artificial intelligence (XAI) for 3D data, significant challenges remain. First, the “black box” nature of the models in this field, whose decision-making processes are opaque makes scrutiny difficult [7], while the “curse of dimensionality” in 3D the complex and varying nature of 3D input formats, such as volumetric scans, voxel grids, and point clouds, poses additional challenges for designing unified and reliable explainability methods [5]. This leads to a lack of explainability, which is particularly problematic in high-stakes domains such as healthcare, where accountability, trust, and transparency are essential for clinical adoption [8,9,10]. Current research mostly focuses on specific tasks or model architectures, limiting the applicability of proposed solutions to only certain domains of the field [8], which can raise concerns about how well these explanations translate to diverse real-world scenarios.

The evaluation of XAI methods often relies on subjective, human-centered assessments, such as visual inspection of saliency maps or heatmaps, rather than objective, standardized benchmarks [11]. While frameworks exist for collecting human feedback on explanation quality, these approaches are inherently subjective and may vary significantly depending on the user’s expertise or expectations. The lack of unified, quantitative metrics for explanation quality leads to inconsistent reporting and makes it difficult to compare methods across studies [12,13]. As a result, practitioners and stakeholders are left without clear guidance on which XAI techniques are most reliable or trustworthy in practice [14].

In this paper, we address these challenges by proposing and implementing a standardized benchmarking framework for evaluating the quality of 3D XAI methods across multiple application domains. In this work, the term “domain” refers to distinct real-world problem areas characterized by differing input modalities and learning tasks. In this paper, specifically, we cover medical imaging, 3D object recognition, and 3D scene understanding. This framework combines both post hoc attribution methods and intrinsic explainability, which are then evaluated on explanation quality using correctness, completeness, and compactness metrics. This provides the first systematic comparison of how explanation methods behave across heterogeneous 3D modalities.

In this paper, we evaluate 3D XAI methods across three representative domains: medical imaging with volumetric chest CT for COVID-19 severity prediction (MosMed), synthetic 3D object recognition using voxelized CAD models (ModelNet40), and real-world point cloud classification in cluttered scenes (ScanObjectNN) [15,16,17].

The different structural and semantic properties of each domain and dataset enable the assessment of a wide variety of inputs. We benchmark both post hoc explainability techniques, such as 3D Grad-CAM [18] and gradient-based Captum methods (Integrated Gradients [19], Saliency, Occlusion), as well as intrinsic models like ResAttNet [20] that incorporate attention directly into the architecture.

We evaluate MosMed using both pretrained and intrinsic models. We voxelized ModelNet40 data, originally in point cloud format, to allow compatibility with 3D CNN-based methods, and analyzed using VoxNet [21] and ResAttNet [20]. We tested ScanObjectNN using a PointNet++ classifier [2]. The intrinsic ResAttNet model was not applied to ScanObjectNN due to its architectural incompatibility with unordered point clouds and poor performance when trained on voxelized versions of this dataset (resulting in classification accuracy plateauing at approximately 53%, making it unsuitable for further intrinsic evaluation). For each method, attribution maps were evaluated using standardized quantitative metrics: Correctness, Completeness, and Compactness [22].

### Positioning of This Paper and Contributions

Although several recent works have proposed XAI methods for handling 3D data, most are confined to individual datasets or domains, and few offer cross-domain comparisons under a unified protocol. The Ren et al.’s visual analytics framework  [23] demonstrates promising cross-modal adaptation but lacks medical volume support, while Cheung et al.’s clinical validation [24] focuses exclusively on cardiac CT. While existing benchmarks like Co-12 [12] provide multi-dimensional frameworks for 2D XAI, our framework aims to distinguish itself with its unified, cross-modal application designed for the complex challenges of 3D data. Unlike previous studies that focus on single modalities, this work provides a direct comparison between intrinsic and post hoc methods across volumetric, voxelized, and point-cloud representations and employs nonparametric statistical tests within a consistent pipeline. Furthermore, we employ non-parametric statistical analysis to measure the magnitude of differences. This study aims to develop a benchmarking framework that:

- Applies multiple post hoc methods and intrinsic models across three distinct 3D domains.
- Relies on pretrained models to ensure realistic, application-relevant evaluation conditions.
- Introduces standardized, quantitative evaluation metrics (Correctness, Completeness, Compactness) [12,22].
- Compares explainability results using non-parametric statistical tests (Kruskal–Wallis, Mann–Whitney U) with exact permutation implementations to control Type I error to ensure robust validation, addressing the lack of statistical rigor noted in recent surveys [12].

Addressing the current gaps, our key differentiators from prior work include the following: (i) cross-modal robustness tests beyond single-dataset evaluations, (ii) a unified cross-modality implementation of XAI metrics, and (iii) statistical validation of metric consistency across 10+ runs. By aligning explainability evaluation across domains and methods, we aim to move toward a more standardized, interpretable, and reliable application of 3D deep learning [4].

Our contributions, therefore, include the following:

- A cross-domain benchmarking framework for evaluating 3D XAI methods that operates consistently across volumetric CT scans, voxelized CAD models, and real-world point clouds by ensuring reproducibility, cross-domain compatibility, and scalability, thereby enabling fair and extensible evaluation of 3D explainability methods.
- A unified implementation of quantitative evaluation metrics, i.e., Correctness, Completeness, and Compactness, applied consistently across all datasets and modalities to systematically evaluate intrinsic (attention-based) and post hoc attribution methods.
- A statistical analysis of method performance on Correctness, Completeness, and Compactness using Kruskal–Wallis and Mann–Whitney U tests, highlighting significant differences in explanation quality.

Together, these contributions lay the foundation for a unified and scalable benchmarking approach that supports transparent and fair evaluation of explainability methods across 3D domains.

## 2. Related Work

While substantial progress has been made in explainability for 2D computer vision models [18,25], the transition to 3D data remains less explored [5]. Recent surveys confirm that 3D XAI faces unique scalability and evaluation challenges not present in 2D domains [5]. The unique geometric, structural, and spatial properties of 3D data—such as point clouds, voxel grids, and volumetric medical scans—complicate the development of general-purpose explainability techniques. In particular, the absence of solid benchmarking frameworks and standardized evaluation metrics for 3D explainability makes it difficult to compare methods or assess their utility across domains [5,14].

We review existing research on 3D explainability, grouped into two broad categories: intrinsic and post hoc methods. We also discuss the role of pretrained models in enabling explainability pipelines and highlight the need for quantitative evaluation strategies.

### 2.1. Intrinsic and Post Hoc Explainability Methods

Explainability methods can be categorized into two major paradigms: intrinsic and post hoc.

#### Intrinsic Explainability

Intrinsic methods embed explainability directly into the model architecture, enabling the network to generate interpretable outputs during training and inference. One such approach is the Residual Attention Network (ResAttNet), which introduces trainable attention masks to modulate feature activations and highlight salient input regions [20,26]. These attention modules are particularly well-suited for tasks involving structured spatial data, such as volumetric medical scans.

Beyond attention mechanisms, recent intrinsic approaches include prototype-based networks that learn medically interpretable features through case-based reasoning [27], self-explaining 3D ResNets that generate clinician-aligned heatmaps without post-processing [24], and mesh-based methods like ExMeshCNN  [28], which incorporate geometric topology into attention mechanisms for 3D shape analysis. However, most intrinsic architectures remain highly domain specific, with recent studies showing that geometric invariance is a key challenge for cross-domain generalization [5].

Post Hoc Explainability

Post hoc methods produce explanations after the model has been trained. It can be model-agnostic or architecture-specific, and typically generates attribution maps that highlight which input regions most influenced the model’s output [4,18,22,29].

Despite their utility, critical limitations hinder the application of post hoc methods in 3D contexts. For instance, they often suffer from gradient instability in sparse point clouds due to discontinuous neighborhoods [5,23]. Additionally, visual clutter is common in volumetric attributions, and overlap significantly reduces human interpretability [23]. These methods can also incur substantial computational expense, sometimes resulting in processing times much longer than those of their 2D counterparts [24]. Furthermore, explanation quality may exhibit significant viewpoint dependency, varying considerably across viewpoints [23].

Perturbation-based methods, such as SHAP and LIME-3D, while powerful, are constrained by high computational expense [30]. This renders them less scalable for high-resolution 3D data and, consequently, less suitable for multi-domain benchmarking.

### 2.2. Transfer Learning and Pretrained Models

Pretrained models play a central role in XAI benchmarking studies. Due to the computational cost and data scarcity involved in training 3D deep learning models from scratch, transfer learning is a convenient solution for conducting XAI research more quickly and effectively. However, transfer learning across domains and modalities introduces some challenges, such as domain adaptation issues [31], which include:

- Architectural and representation mismatch across volumetric, voxelized, and point cloud data, which prevents a single intrinsic model from being applied uniformly across all domains.
- Modality-specific pre-processing requirements (resampling, voxelization, point normalization) that introduce additional sources of variability and complicate fair cross-domain comparison of explainability methods.
- Domain-dependent behavior of XAI metrics, with Correctness, Completeness, and Compactness favoring different methods in medical CT versus voxelized CAD and point clouds, limiting direct transfer of conclusions across domains, showing high inter-rater disagreement [31].

### 2.3. Evaluation of 3D Explainability Techniques

In 2D XAI, evaluation methods such as insertion/deletion curves or Intersection over Union (IoU) with human-annotated ground truth are common. However, such strategies are often unsuitable or unavailable in 3D XAI due to the spatial complexity and lack of semantic annotation. Medical annotation studies reveal longer labeling times versus 2D, often disagreement [31].

The Co-12 framework, introduced by Nauta et al. [12], proposes a multi-dimensional approach to XAI evaluation, covering fidelity, stability, and usability dimensions.

Recent benchmarks highlight various trade-offs in XAI method performance. For instance, optimizing for compactness has been shown to reduce completeness across modalities [12]. Such inherent tradeoffs underscore the critical importance of evaluating explanation quality through multiple, complementary metrics to gain a broad and nuanced understanding.

## 3. Methodology

Our proposed framework comprises data preprocessing, domain-specific model training, extraction of an explainability map, and metric-based building blocks. The framework aims to provide cross-domain comparability of benchmarking and to facilitate consistent analysis of explainability methods under varying conditions of data fidelity, noise, and task complexity.

**Figure 1:**
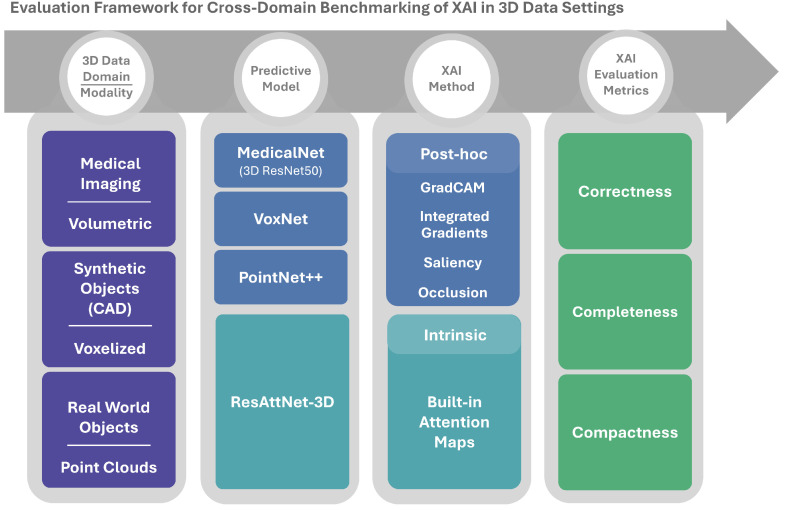
Overview of the cross-domain 3D XAI benchmarking framework.

Figure 1 illustrates the main components of the framework. From left to right, it illustrates: (i) the 3D data domains and modalities, (ii) the predictive models used for each domain, (iii) the intrinsic and post hoc XAI methods, and (iv) the quantitative evaluation metrics. The following subsections describe these components in more detail.

### 3.1. Three-Dimensional Data Domains and Learning Tasks

The benchmark covers three representative 3D data domains, each with its own geometric representation and learning task. Together, these three datasets cover a broad spectrum of 3D modalities, i.e., volumetric, voxelized, and point cloud, and allow for a cross-domain evaluation of 3D modality explanations.

- MosMed–Volumetric CT Classification [15] MosMed contains chest CT scans labeled with COVID-19 severity (CT-0–CT-4). Due to extreme imbalance in the CT-4 class, our study focuses on a four-class setting (CT-0–CT-3). Scans are resampled to a standardized volume of 64×128×128 and intensity-normalized. This domain reflects a high-stakes medical imaging scenario, where interpretability is essential for clinical adoption and for verifying that explanations align with relevant anatomical structures.
- ModelNet40–Voxelized CAD Model Classification [17] ModelNet40 consists of over 12,000 3D CAD models from 40 object categories. In our framework, polygonal meshes are voxelized into $32^{3}$ binary occupancy grids. This resolution was selected to maintain architectural compatibility with the VoxNet framework [21] and to ensure a balance between predictive accuracy and computational efficiency during the explainability extraction process. While higher resolutions can capture finer geometric details, the $32^{3}$ grid remains sufficient for the model to distinguish between the 40 object categories while keeping the input dimensionality manageable for intensive perturbation-based XAI metrics. This provides a structured, grid-based representation that complements the dense medical volumes and allows us to study explainability in synthetic object recognition tasks.
- ScanObjectNN–Real-World Point Cloud Classification [16] ScanObjectNN offers real-world object point clouds acquired from RGB-D scene constructions derived from the SceneNN and ScanNet datasets. In cluttered indoor scenes. Each point cloud is downsampled to 1024 points and normalized to a unit sphere. Compared with MosMed and ModelNet40, this domain is inherently noisy and sparse, and thus well suited for examining how XAI methods behave on unstructured 3D data.

### 3.2. Predictive Models

We benchmark the performance of two types of 3D predictive models: pretrained architectures used with post hoc explainability and an attention-based model providing intrinsic interpretability. Each model is chosen to match the structure of its input data:

- MedicalNet (ResNet-50): For MosMed, we use the 3D ResNet-50 architecture from the MedicalNet framework  [31]. The final classification layer is adapted to predict four COVID-19 severity levels (CT-0–CT-3). Input volumes are single-channel tensors of shape 1×64×128×128.
- VoxNet: For ModelNet40 voxel grids (1×32×32×32), we use VoxNet [21], a compact 3D CNN designed for voxelized object recognition.
- PointNet++: For ScanObjectNN point clouds, we use PointNet++ [2], which applies hierarchical set abstraction to capture local and global geometric patterns in unstructured point data.
- ResAttNet-3D: To study intrinsic interpretability, we implement ResAttNet-3D [26], an attention-based 3D CNN. The model is trained on MosMed and ModelNet40 using the same preprocessed volumes and voxel grids as above. Its attention modules generate spatial masks that highlight regions the network focuses on during inference.

### 3.3. Explainability Methods

To interpret model predictions, the benchmark combines post hoc attribution methods applied to pretrained models and intrinsic attention maps from ResAttNet-3D. All attribution maps are min–max normalized to [0,1] and stored in standardized NumPy-based formats for subsequent evaluation.

#### 3.3.1. Post Hoc Attribution Methods

Four post hoc methods are used across the three domains:

- Grad-CAM generates class-discriminative heatmaps by weighting the feature maps of a target convolutional layer using the gradients of the predicted class score. For volumetric and voxel inputs, the gradients are backpropagated through the final 3D convolutional layer [18]. For PointNet++, we adapt this method by capturing features and gradients from the second set abstraction (sa2) layer and using Open3D’s KDTree for nearest-neighbor interpolation to upsample the heatmap to the original point cloud resolution, inspired by the implementation of Fragjacker/Pointcloud-grad-CAM [32].
- Saliency Maps highlight voxels or points whose slight changes most affect the predicted class score. This method computes the gradient of the score with respect to the input tensor, takes its absolute value, and aggregates it using the L2 norm for visualization. For point clouds, the L2 norm is computed across channels per point using  [33], and the approach is validated against sanity checks for saliency [34].
- Integrated Gradients accumulate gradients along a path from a baseline input to the actual input, providing a more stable attribution  [19].
- Occlusion masks local regions of the input and measures the resulting change in prediction confidence. The method slides a fixed-size 3D window (occlusion patch) across the input volume, replacing covered voxels/points with baseline values (e.g., mean intensity or zero). The resulting prediction difference quantifies region importance: significant score drops indicate critical features for model decisions [35].

For PointNet++, Grad-CAM activations computed at the set abstraction layer are projected back to the input point cloud using KDTree-based nearest-neighbor interpolation. To qualitatively validate this mapping, interpolated Grad-CAM attributions were visually compared with raw point-wise saliency maps, showing consistent localization of dominant geometric structures while exhibiting reduced noise.

The chosen post hoc methods provide complementary perspectives on model behavior while balancing computational efficiency and architectural compatibility. Grad-CAM delivers class-discriminative explanations but requires convolutional layers for activation mapping [18], making it unsuitable for non-convolutional architectures. Integrated Gradients offer axiomatic soundness [19] but incur high computational costs. Occlusion provides intuitive perturbation-based insights but scales poorly with input dimensionality [36]. Surrogate-based methods such as LIME [30] were excluded not only due to prohibitive computational demands when generating local surrogate models for large 3D inputs, but also because their explanations depend on an auxiliary model approximation rather than directly reflecting the behavior of the original network, which complicates controlled comparison under a unified perturbation-based evaluation protocol. The implementations prioritize the following: (1) architectural compatibility (convolutional layers for Grad-CAM, generic input handling for Saliency/IG); (2) computational feasibility (stride optimization, GPU fallback); and (3) modality-specific adaptations (KDTree upsampling for point clouds [32], anatomical scaling for medical volumes). This balanced approach enables comprehensive benchmarking across diverse 3D data types while respecting practical constraints.

#### 3.3.2. Intrinsic Attention Maps

The ResAttNet-3D architecture embeds attention mechanisms that generate interpretable maps as part of the model’s forward pass. These attention maps highlight regions the model focuses on during inference, adapting from low-level feature detection (e.g., color, texture) to high-level semantic understanding (e.g., object parts) as demonstrated in Wang et al.’s hierarchical attention framework [20].

### 3.4. Evaluation Metrics and Protocol

The quality of explanations is evaluated using three quantitative metrics that capture complementary aspects of interpretability: correctness, completeness, and compactness which are generalized in the systematic review of XAI evaluation conducted by Nauta et al. [12]. These metrics are chosen because they generalize across volumetric, voxelized, and point-cloud data and do not require ground-truth explanation labels. Our evaluation protocol incorporates both correctly and incorrectly classified samples to reflect real-world model behavior. For incorrect predictions, the metrics assess the quality of the explanation relative to the model’s actual decision (e.g., why a ‘table’ was misidentified as a ‘chair’) rather than comparing it to a ground-truth label. Furthermore, to address baseline sensitivity in AOPC/AUPC computations, we utilize modality-specific neutral states. Specifically, we define these as zero-value baselines for geometric data (ModelNet40 and ScanObjectNN) representing the total absence of structural features, and as mean-intensity baselines for volumetric CT data (MosMed). By calculating the area under or over the curve across multiple perturbation steps, the framework focuses on the overall trend in the model’s confidence. This is to ensure that the resulting scores reflect the method’s performance and are not sensitive to a single baseline value. Hence, this pipeline automates scoring, statistical analysis, and reporting across diverse 3D modalities.

#### 3.4.1. Correctness (AOPC)

**Figure 2:**
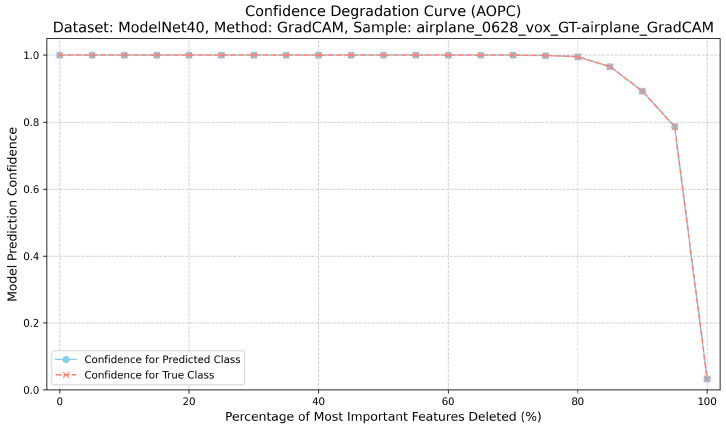
Confidence degradation curve (AOPC) for ModelNet40 ‘airplane’ sample using GradCAM, demonstrating causal relevance assessment.

Correctness evaluates the causal relevance of highlighted regions. For each explanation, we iteratively remove the top-k% most attributed voxels or points and record the model’s confidence for the target class [22]. This procedure for calculating the AOPC score is detailed in Algorithm 1. The Area Over the Perturbation Curve (AOPC) summarizes the resulting confidence drop: higher AOPC values indicate that the explanation identifies regions that are truly important for the prediction. For its implementation, features are perturbed by setting their values to the mean, zero, or a blurred intensity, and the AOPC is then computed from the confidence curve using numerical integration. A higher AOPC value indicates stronger causal relevance [12] (Figure 2).
**Algorithm 1** AOPC Computation Procedure
  1: **procedure**
ComputeAOPC($f,x,a,y_{pred},K$)

**Require:** model *f*, input *x*, attribution map *a*, predicted class label $y_{pred}$, steps *K*

**Ensure:** AOPC score

  2:        $conf_{init}\leftarrow f\left(x\right)$ for class $y_{pred}$

  3:        confidences ← empty list

  4:        indices ← sort *a* by importance (highest first)

  5:        **for** k=1 to *K* **do**

  6:              fraction ←k/K (e.g., 0.1, 0.2, …, 1.0)

  7:           $x_{perturbed}\leftarrow x$ with top fraction removed (set to neutral value)

  8:              $conf_{k}\leftarrow f\left(x_{perturbed}\right)$ for class $y_{pred}$

  9:              confidences.append($conf_{k}$)

10:        **end for**

11:        $drop\leftarrow [conf_{init}-c\;\mathrm{for}\;\mathrm{each}\;c\;\mathrm{in}\;\mathrm{confidences}]$

12:        AOPC ← area under drop curve (numerical integration)

13:        **return** AOPC

14:** end procedure**


#### 3.4.2. Completeness (AUPC)

**Figure 3:**
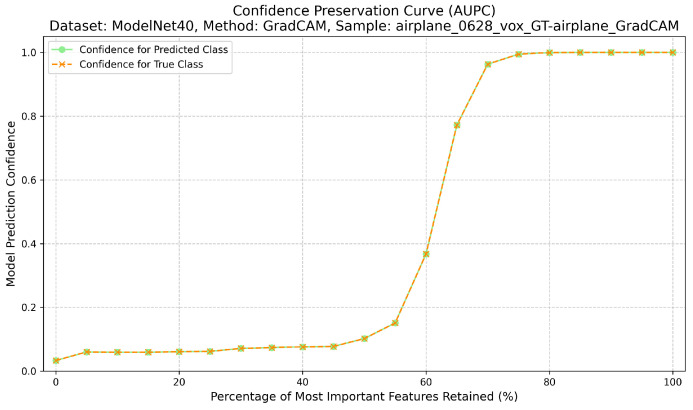
Confidence preservation curve (AUPC) showing explanation sufficiency.

Completeness measures the sufficiency of the explanation. Here, only the top-k% most attributed regions are retained, while the rest of the input is masked with neutral values. The Area Under the Preservation Curve (AUPC) aggregates the resulting confidence values; higher AUPC scores indicate that the explanation captures enough information to sustain the prediction when non-attributed regions are removed [12] (Figure 3). The calculation of the AUPC metric, which quantifies this sufficiency, is outlined in Algorithm 2.
**Algorithm 2** AUPC–Area Under Preservation Curve (Completeness)
  1: **procedure**
ComputeAUPC($f,x,a,y_{pred},K$)

**Require:** model *f*, input *x*, attribution map *a*, predicted class label $y_{pred}$, steps *K*

**Ensure:** AUPC score

  2:        confidences ← [0.0] (baseline with empty input)

  3:        indices ← sort *a* by importance (highest first)

  4:        **for** k=1 to *K* **do**

  5:              fraction ←k/K

  6:              $x_{preserved}\leftarrow x$ with only top fraction kept

  7:              $conf_{k}\leftarrow f\left(x_{preserved}\right)$ for class $y_{pred}$

  8:              confidences.append($conf_{k}$)

  9:        **end for**

10:        AUPC ← area under confidences curve

11:        **return** AUPC

12: **end procedure**


#### 3.4.3. Compactness

Compactness quantifies the spatial focus of explanations by measuring how concentrated the attribution mass is within the input [12]. This metric is calculated by measuring the ratio of highlighted elements as shown in Algorithm 3. Fixed and adaptive thresholds are used to compute ratios between highlighted voxels/points and the total input size, yielding a measure of how parsimoniously the explanation localizes relevant evidence. Therefore, lower fixed threshold values (e.g., 0.2414) indicate focused explanations, while higher adaptive threshold values suggest concentrated attribution.
**Algorithm 3** Compactness–Spatial Concentration Metric
  1: **procedure**
ComputeCompactness(a,x,t)

**Require:** attribution map *a*, input *x*, threshold values *t* (e.g., 0.5, 0.7, 0.9)

**Ensure:** compactness scores

  2:        $n_{nonzero}\leftarrow$ count of non-zero values in *x*

  3:        results ← empty dictionary

  4:        **for** each threshold *t* **do**

  5:              $n_{important}\leftarrow$ count of values in *a* where $a_{i}\geq t$

  6:              ratio $\leftarrow n_{important}/n_{nonzero}$

  7:              results[“compactness_t” + *t*] ← ratio

  8:        **end for**

  9:        **return** results

10: **end procedure**


#### 3.4.4. Evaluation Pipeline and Cross-Domain Consistency

For each dataset and model configuration, the evaluation pipeline loads precomputed attribution maps from selected up to five (at most) samples per class, including both correctly and incorrectly classified instances. Then, for these samples, it generates explainability maps using the respective XAI methods under study and later averages our metrics across these samples to balance the dataset for the XAI evaluation metrics. Following these preparatory steps, the pipeline computes the AOPC, AUPC, and Compactness scores for all selected samples. This approach enabled detailed per-class and per-sample analysis of explainability quality.

Although intrinsic attention mechanisms and post hoc attribution methods differ in how explanations are generated, both produce spatial importance maps that assign relevance values to voxels or points. In this study, evaluation is performed at the level of explanation outputs rather than at the level of generation mechanisms. Perturbation-based metrics assess how these importance maps reflect the model’s decision behavior, regardless of whether they are derived from intrinsic or post hoc methods. Treating both explanation types as functional equivalents at the evaluation stage enables a unified and consistent benchmarking framework across models and data modalities.

Additionally, to ensure robust comparison, we used non-parametric statistical tests that do not assume a specific distribution [37]. We employed the Kruskal–Wallis for multi-group comparisons, followed by Bonferroni-corrected Mann–Whitney U tests, applied per dataset–metric combination to assess whether performance differences between XAI methods are statistically significant for pairwise analysis. To control for multiple comparisons, the [38] recommends Bonferroni correction as a straightforward approach to managing Type I error across multiple hypothesis tests. Additionally, effect sizes (Cohen’s *r*) were calculated from the Z-score of the Mann–Whitney U statistic to distinguish significance from just probability.

## 4. Experimental Results

To ensure a fair and reproducible comparison of explainability methods across heterogeneous 3D learning tasks, all experiments were conducted using a unified training and evaluation pipeline. The benchmarking framework described in Section 3 standardizes data preprocessing, predictive model training, explainability map generation, and metric computation across the three selected domains; the specific hyperparameters and configurations for each predictive model are summarized in Table 1.

For explainability, post hoc XAI methods (Grad-CAM, Saliency, Integrated Gradients, and Occlusion) were applied to all predictive models, with modality-specific adaptations to account for differences between volumetric, voxel, and point-cloud representations. Layer selection, baselines, step sizes, and occlusion window parameters are detailed in Table 2. Intrinsic explanations were obtained from the attention modules of the ResAttNet-3D model, with attention masks appropriately upsampled for each modality.

### 4.1. Predictive Model Performance

Each predictive model was evaluated on its corresponding 3D domain using accuracy, precision, recall, and F1-score metrics. Table 3 summarizes the macro- and weighted/overall averages from the classification reports.

On the MosMed dataset, MedicalNet (ResNet-50) achieved high predictive reliability with a weighted F1-score of 0.9613, indicating balanced discrimination across the four COVID-19 severity levels. Both macro and weighted metrics exceeded 0.96, confirming the model’s robustness and suitability as a foundation for post hoc explainability analyses. The intrinsic ResAttNet-3D model yielded slightly lower weighted performance (F1 = 0.9188) yet demonstrated strong interpretability potential. Its built-in spatial attention modules effectively separated severity categories CT-0 to CT-3, achieving 91.9% test accuracy while correctly identifying mild and severe cases with high precision. Misclassifications primarily occurred between CT-1 and CT-2, consistent with their visual similarity in chest CT imagery.

For ScanObjectNN, the pretrained PointNet++ model attained an overall test accuracy of 84.5% and a weighted F1-score of 0.8442, showing reliable generalization across noisy, real-world point clouds. Classes with distinctive geometric structures (e.g., chair, display, sofa) achieved F1-scores > 0.93, while similar or underrepresented categories (e.g., desk, pillow, sink) were less consistent due to class imbalance and occlusion noise.

On the ModelNet40 benchmark, VoxNet reached a test accuracy of 81.2% with a weighted F1 of 0.8104, providing a strong voxel-based baseline. The intrinsic ResAttNet-3D outperformed VoxNet, reaching 83.8% accuracy and a weighted F1 of 0.8389. Attention integration improved feature discrimination for complex 3D shapes, such as a car, a sofa, and a toilet, though confusion persisted for visually similar small objects, such as a flower pot or a cup. These results collectively confirm that all predictive models achieved a sufficient level of performance and reliability to serve as stable backbones for subsequent explainability benchmarking across modalities.

### 4.2. Quantitative Evaluation of XAI

For each dataset, five samples per class were selected, balanced between correctly and incorrectly classified instances. This sampling strategy provided a representative distribution of predictions for each model–dataset pair, while keeping computational cost feasible given the 3D data volume. Each sample underwent normalization and, when necessary, interpolation to a unified spatial resolution of 112 × 112 × 112 for volumetric and voxelized inputs, and 1024 points for point clouds. All results were averaged per dataset and visualized as boxplots to illustrate distributional trends across methods.

All results were computed on the test sets of MosMed, ModelNet40, and ScanObjectNN using identical metric definitions to ensure cross-domain comparability.

#### 4.2.1. Correctness Results

**Figure 4:**
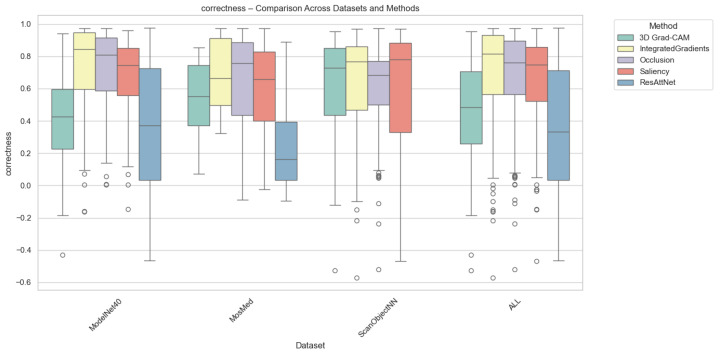
Correctness comparison across datasets and XAI methods. Individual circles represent outliers, defined as data points falling beyond 1.5 times the interquartile range from the box edges.

Correctness evaluates how strongly the model’s confidence decreases when top-attributed regions are removed. Grad-CAM consistently achieves the highest correctness across all datasets, confirming its ability to capture class-relevant regions that directly influence prediction confidence. Integrated Gradients and Saliency follow with moderate correctness, while Occlusion exhibits the lowest scores due to its patch-based sensitivity. As can be seen from Figure 4, for MosMed, Grad-CAM explanations led to the largest confidence drops (0.48), indicating faithful localization of diagnostically important lung areas. ResAttNet-3D’s intrinsic attention achieved slightly lower correctness (0.42), reflecting partial alignment with decision-critical features. Across ModelNet40 and ScanObjectNN, Grad-CAM retained similar superiority, while Integrated Gradients achieved correctness values around 0.35–0.40, consistent with its smoother attribution diffusion.

#### 4.2.2. Completeness Results

**Figure 5:**
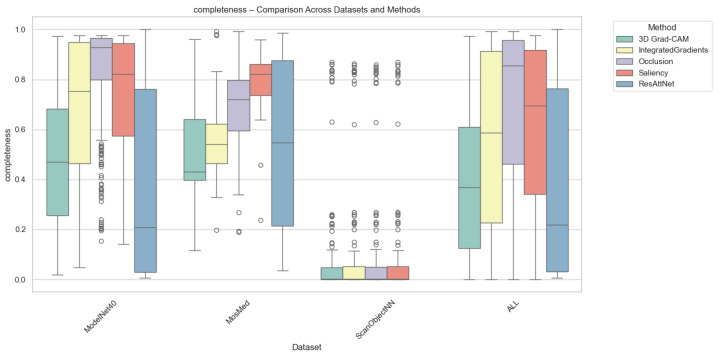
Completeness comparison across datasets and XAI methods. Individual circles indicate statistical outliers detected via the Interquartile range (IQR) method.

Completeness quantifies how well an explanation preserves the model’s prediction confidence when only the top-attributed regions are retained. As depicted in Figure 5, Grad-CAM again achieved the highest completeness for MosMed and ModelNet40, demonstrating that highlighted regions contain sufficient information for maintaining prediction fidelity. In contrast, Integrated Gradients and Saliency methods produced lower completeness scores, suggesting over-attribution to irrelevant regions. Occlusion yielded highly variable completeness scores, which were sensitive to patch size and structural complexity.

On average, completeness values were highest for Grad-CAM (0.72 for MosMed, 0.75 for ModelNet40) and slightly lower for intrinsic attention (0.69), suggesting that built-in attention mechanisms can approximate the post hoc method’s explanatory power. For ScanObjectNN, all methods achieved lower completeness (0.55–0.60) due to increased data irregularity and occlusion sensitivity.

#### 4.2.3. Compactness Results

Compactness was computed in two complementary variants, i.e., (i) Adaptive Compactness (Top 10%), which evaluates attribution density among the top 10% most salient voxels or points, and (ii) Fixed-Threshold Compactness (t = 0.5), which measures the proportion of activations exceeding an absolute normalized threshold.

It is noted that in the adaptive variant of the cross-domain evaluation, some instances exhibit exceptionally high values. These instances occur when the attribution mass is concentrated in significantly fewer voxels than the threshold requires, which results in very high scores.

Outlier values were excluded to ensure numerical stability and comparability. This was done using the Interquartile Range method (IQR) for each method-dataset pair. Samples falling outside the bounds of [Q1−1.5×IQR,Q3+1.5×IQR] were removed. While this filtering prevents excess noise from skewing the overall trends, we acknowledge that the resulting scores reflect method performance under stable conditions and may not fully capture the unpredictable spikes in sensitivity that gradient-based methods often exhibit when processing sparse 3D data structures.

**Figure 6:**
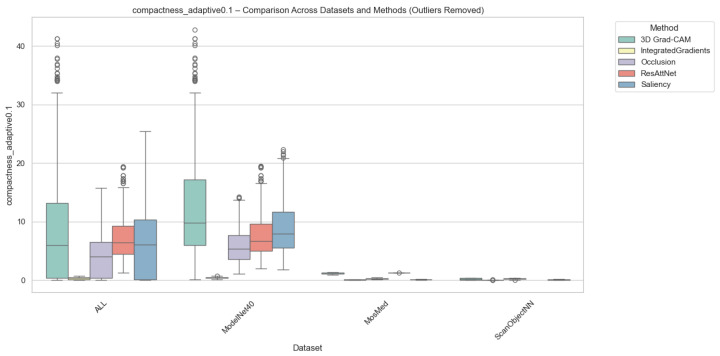
Compactness (Adaptive @ 0.1) comparison across datasets and methods with outliers removed. Higher scores indicate stronger localization among the most salient voxels or points. Note: Circles represent remaining individual samples that exceed the whisker boundaries.

**Figure 7:**
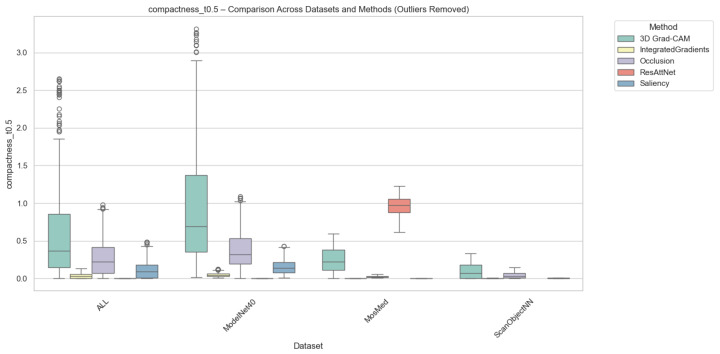
Compactness (Fixed @ t=0.5) comparison across datasets and methods with outliers removed. Higher scores indicate more spatially focused attributions above the fixed threshold. Individual circles denote outlier instances outside the 1.5 × IQR range.

As illustrated in Figure 6 and Figure 7, Occlusion yielded the most compact attributions across all datasets, which achieves mean compactness values between 0.31–0.35. This reflects its inherently localized perturbation mechanism, which highlights small yet highly discriminative regions. Grad-CAM and ResAttNet-3D attention maps yielded moderately compact results (0.28–0.30), balancing local specificity with spatial coverage. In contrast, Saliency and Integrated Gradients tended to produce more diffused activations, resulting in lower compactness (0.22–0.25) across all modalities.

### 4.3. Statistical Significance Analysis

To evaluate whether the observed differences in explanation quality across both intrinsic and post hoc methods are statistically significant, we conducted non-parametric tests on datasets. Five methods were compared: four post hoc (3D Grad-CAM, Integrated Gradients, Occlusion, Saliency) and one intrinsic (ResAttNet). The evaluation focuses on the standardized benchmarking metrics: Correctness, Completeness, and Compactness (fixed threshold at t=0.5).

We first applied the Kruskal–Wallis H-test to detect overall group differences across methods for each metric. Upon significance, pairwise Mann–Whitney U tests were performed to identify specific method pairs with significant differences. All *p*-values were corrected using the Bonferroni method, and effect sizes were calculated as $\eta^{2}$ (Kruskal–Wallis) and Cohen’s *r* (Mann–Whitney). This approach was selected due to the non-normal, heteroscedastic nature of saliency-based distributions, for which parametric ANOVA assumptions were not met.

#### 4.3.1. Kruskal–Wallis Test

Table 4 summarizes the Kruskal–Wallis test results across datasets and metrics. All three metrics for MosMed showed statistically significant differences (p<0.05), with the largest effect size observed for Compactness ($\eta^{2}=0.895$). Correctness ($\eta^{2}=0.303$) and Completeness ($\eta^{2}=0.113$) also demonstrated medium-to-large effects, reflecting substantial variability in causal fidelity and information sufficiency among methods. In ScanObjectNN, significant effects were found for Correctness and Compactness, though with smaller effect sizes, suggesting moderate differentiation between post hoc methods. Completeness did not yield statistically significant differences, consistent with the more uniform attribution coverage across samples. For ModelNet40, all metrics indicated strong significance and large effect sizes, confirming that differences in explanation quality were robust and consistent in the shape-classification domain.

These results collectively indicate that compactness is the most discriminative metric across all datasets, while correctness and completeness reflect model- and dataset-specific behavior. The consistent statistical significance across datasets supports the robustness of the evaluation protocol and highlights that explainability methods produce distinctly different attribution characteristics.

#### 4.3.2. Mann–Whitney U Pairwise Test

Pairwise Mann–Whitney U tests were subsequently performed to identify specific method-level differences. The Bonferroni-corrected results in Table 5 show that, in MosMed, the intrinsic ResAttNet method significantly differed from all post hoc approaches in correctness (p<0.005), with large effect sizes (r>0.5). In contrast, differences among post hoc methods were generally non-significant, which shows comparable fidelity in their attributions.

In ScanObjectNN, significant pairwise differences were primarily observed in compactness, particularly between Integrated Gradients, Occlusion, and Saliency, with large effect sizes confirming their differing spatial aggregation patterns. For ModelNet40, nearly all pairwise comparisons were significant, indicating highly distinct explanation behaviors across methods, consistent with the strong Kruskal–Wallis effects.

The Mann–Whitney results confirm that all metrics, particularly compactness, produce statistically distinct outcomes across explainability methods. In agreement with the Kruskal–Wallis test, post hoc methods generally outperform intrinsic attention maps in correctness and completeness, whereas intrinsic maps exhibit stronger compactness.

## 5. Discussion on Cross-Domain Trends

Table 6 synthesizes all quantitative results across the three datasets (MosMed, ScanObjectNN, and ModelNet40) and five explainability methods (Grad-CAM, Integrated Gradients, Occlusion, Saliency, and intrinsic ResAttNet attention maps). It reports the mean values of Correctness, Completeness, and Compactness under adaptive (a=0.1) and fixed (t=0.5) thresholds. This unified view allows identifying consistent behavioral patterns and anomalies across data modalities and architectures.

Across all domains, Integrated Gradients (IGs) and Occlusion consistently achieved the highest Correctness and Completeness values, indicating stronger causal alignment and information sufficiency. IG achieved the most faithful attributions but tended to produce spatially diffuse explanations, whereas Occlusion provided a balanced trade-off between faithfulness and compactness. Saliency often achieved strong completeness, suggesting broader activation coverage, but also generated less localized attributions.

ResAttNet’s intrinsic attention maps were systematically the most compact, especially in volumetric and voxelized data, confirming their superior spatial focus. However, they suffered from low correctness and completeness, implying that their attention was sharply localized but not necessarily aligned with causal regions. This supports the existence of a faithfulness–compactness trade-off observed across all datasets.

Grad-CAM exhibited domain-dependent behavior: it was moderately successful for structured, grid-based data such as ModelNet40 but struggled with irregular volumetric or point-based inputs (MosMed and ScanObjectNN), where gradient saturation led to weak saliency localization. Outlier compactness values in ModelNet40 (e.g., Grad-CAM and Occlusion) likely originate from sparsity and instability in adaptive compactness metrics.

Across datasets, faithfulness-oriented methods (IG, Occlusion) performed best on correctness and completeness, whereas localization-oriented methods (ResAttNet) excelled in compactness. This duality was consistent with the statistical findings: while all metrics captured distinct interpretability dimensions, compactness emerged as the most discriminative measure across modalities. The results underscore that 3D explainability cannot be generalized across domains without considering both architectural dependencies and data modality structures.

Together, these findings affirm the need for multi-metric evaluation frameworks that balance spatial precision with causal faithfulness, ensuring fair and interpretable comparisons of 3D XAI methods across medical, geometric, and point-based datasets.

We acknowledge the limitation of using a subset of five samples per class for the explainability evaluation. This constraint was due to the high computational cost of 3D XAI extraction across multiple datasets. However, by aggregating these into a total of approximately 200 samples per dataset and applying non-parametric statistical tests with Bonferroni corrections, we aimed to ensure that the reported trends were statistically robust.

The proposed benchmark does not include intrinsic explainability for point-cloud architectures, as the attention modules of ResAttNet-3D operate on dense convolutional feature maps and are not directly compatible with the set abstraction and aggregation mechanisms of PointNet++. Supporting intrinsic explanations for point-based models would require architectural redesign and is therefore considered beyond the scope of the current study.

## 6. Conclusions and Future Directions

In this paper, we presented a standardized, extensible, and statistically validated framework for benchmarking explainability methods in 3D deep learning. By integrating intrinsic and post hoc approaches within a unified, metric-driven evaluation pipeline, the framework enables reproducible, cross-domain comparison of explainability quality across medical volumetric data (MosMed), voxelized CAD models (ModelNet40), and point-cloud scenes (ScanObjectNN). Three complementary metrics, i.e., Correctness, Completeness, and Compactness, formed the quantitative foundation of this benchmark, which enables systematic evaluation.

Across all experiments, the results revealed clear, statistically significant trade-offs between correctness (i.e., causal faithfulness) and spatial compactness. Post hoc methods, such as Integrated Gradients and Occlusion, consistently demonstrated strong correctness and completeness, whereas intrinsic models, such as ResAttNet, produced highly compact yet less causally faithful attributions. These findings, supported by Kruskal–Wallis and Mann–Whitney tests with robust effect-size analysis, confirm that no single explainability method is universally optimal. Instead, the suitability of an XAI approach depends on the domain’s structural properties and interpretability goals.

While the framework advances the state of quantitative 3D XAI evaluation, several limitations remain. The selection of classification-based datasets was motivated by the need for a consistent evaluation protocol across heterogeneous 3D modalities (i.e., avoiding task-specific metrics required for semantic segmentation). Its current scope covers three representative data modalities and model types, leaving future extensions to additional architectures (e.g., transformers, graph-based networks) and to multimodal inputs. Adaptive compactness proved unstable in certain conditions, underscoring the importance of metric validation and fixed-threshold formulations. Moreover, the absence of expert-annotated ground truth limits direct human-centered evaluation.

Future research may expand this benchmark toward human-in-the-loop and geometry-aware evaluation pipelines that connect quantitative metrics with expert perception. Developing intrinsic explainability for unstructured 3D data, refining causality-aware metrics, and ensuring geometric invariance across transformations remain critical next steps. Together, these directions can strengthen the trustworthiness, explainability, and generalizability of 3D AI systems deployed in clinical and real-world environments.

## Figures and Tables

**Table 1:** Experimental configurations for each predictive model and corresponding dataset.

Dataset	Predictive Model	Input Shape	Classes	Train Test Split	Loss Function	Optimizer	Learning Rate	Epochs	Batch Size
MosMed	MedicalNet (ResNet-50)	1 × 64 × 128 × 128	4	80/20	Cross Entropy	SGD	exp. decay schedule	100	4
	ResAttNet-3D	1 × 64 × 128 × 128	4	80/20	Weighted Cross Entropy	Adam	step decay	100	32
ModelNet40	VoxNet	1 × 32 × 32 × 32	40	80/20	Cross Entropy	Adam	1.00 × 10^4^	50	64
	ResAttNet-3D	1 × 32 × 32 × 32	40	80/20	Weighted Cross Entropy	Adam	step decay	100	32
ScanObjectNN	PointNet++	B × 3 × 1024	15	80/20	Negative Log Likelihood	Adam	0.001	100	32

**Table 2:** Configurations of the explainability methods used in this study. Abbreviations: Med = MedicalNet; VN = VoxNet; PN++ = PointNet++; PC = point cloud; vol = volumetric input; vox = voxelized input; pts = point-based input, N/A = Not Applicable.

Approach	Method	Layer Used	Baseline	Steps/Window	Modality Adaptation
Post Hoc	Grad-CAM	Last Conv (Med, VN) SA2 (PN++)	N/A	N/A	Upsampling: zoom for vol/vox; KDTree for PC
	Saliency	Input gradients	N/A	N/A	Point-wise L2 norm for PC
	Integrated Gradients	Input gradients	Zero	50 steps	Works for all modalities
	Occlusion	Sliding-window perturbation	Mean (vol/vox), Zero (points)	vol: (1, D/8, H/8, W/8); vox: (1,4,4,4); pts: cluster of 10	PC use cluster removal
Intrinsic	ResAttNet-3D Attention Maps	soft2 mask of attention modules	N/A	N/A	Upsampled via trilinear interpolation

**Table 3:** Summary of predictive model performance on the test sets. Values are taken directly from the original classification reports and reported as macro-averaged and weighted/overall Precision, Recall, and F1-score. The ^†^ denotes overall test accuracy values referenced in the text, presented here in decimal format.

Dataset	Model	Macro Avg			Weighted/Overall		
		Precision	Recall	F1-Score	Precision	Recall	F1-Score
MosMed	MedicalNet (ResNet-50)	0.9611	0.9631	0.9618	0.9617	0.9617	0.9613
MosMed	ResAttNet-3D	0.7410	0.7145	0.7270	0.9190 ^†^	0.9189 ^†^	0.9188 ^†^
ScanObjectNN	PointNet++	0.8514	0.8077	0.8199	0.8572	0.8451	0.8442
ModelNet40	VoxNet	0.7436	0.7442	0.7383	0.8160 ^†^	0.8120 ^†^	0.8104 ^†^
ModelNet40	ResAttNet-3D	0.7760	0.7814	0.7759	0.8440	0.8383	0.8389

**Table 4:** Kruskal–Wallis test results across datasets and metrics. Effect size $\eta^{2}$ values are interpreted following Cohen’s convention: 0.01–small, 0.06–medium, 0.14+–large. Significant results (p<0.05) are highlighted in green and Non-Significant results (p≥0.05) are highlighted in red.

Dataset	Metric	H-Statistic	*p*-Value	$\eta^{2}$ (Effect Size)
MosMed	Correctness	49.15	$5.42\times 10^{-10}$	0.303 (Large)
MosMed	Completeness	20.79	$3.5\times 10^{-4}$	0.113 (Medium)
MosMed	Compactness (t=0.5)	137.37	$1.03\times 10^{-28}$	0.895 (Large)
ScanObjectNN	Correctness	8.88	0.031	0.011 (Small)
ScanObjectNN	Completeness	0.009	0.9998	– (Negligible)
ScanObjectNN	Compactness (t=0.5)	179.76	$9.92\times 10^{-39}$	0.327 (Large)
ModelNet40	Correctness	601.06	$9.16\times 10^{-129}$	0.243 (Large)
ModelNet40	Completeness	693.79	$7.70\times 10^{-149}$	0.281 (Large)
ModelNet40	Compactness (t=0.5)	2133.25	<$10^{-300}$	0.868 (Large)

**Table 5:** Pairwise Mann–Whitney *U* test results across datasets and metrics. *p*-values are Bonferroni-corrected; significant comparisons (p<0.005) are highlighted in green. Effect sizes (*r*) follow Cohen’s guideline: 0.1–small, 0.3–medium, 0.5–large.

Dataset	Compared Methods	Metric	Corrected *p*	*r* (Effect Size)
MosMed	ResAttNet vs. IG	Correctness	$2.57\times 10^{-7}$	0.666 (Large)
MosMed	ResAttNet vs. Occlusion	Correctness	$4.08\times 10^{-6}$	0.606 (Large)
MosMed	ResAttNet vs. Saliency	Correctness	$1.58\times 10^{-5}$	0.575 (Large)
MosMed	ResAttNet vs. Grad-CAM	Correctness	$3.1\times 10^{-4}$	0.499 (Medium)
ScanObjectNN	IG vs. Occlusion	Compactness (t=0.5)	$3.40\times 10^{-44}$	0.853 (Large)
ScanObjectNN	Occlusion vs. Saliency	Compactness (t=0.5)	$4.46\times 10^{-38}$	0.790 (Large)
ScanObjectNN	IG vs. Saliency	Compactness (t=0.5)	$7.70\times 10^{-8}$	0.344 (Medium)
ScanObjectNN	Grad-CAM vs. IG	Compactness (t=0.5)	$1.11\times 10^{-3}$	0.226 (Small)
ScanObjectNN	Grad-CAM vs. Saliency	Compactness (t=0.5)	$2.15\times 10^{-3}$	0.216 (Small)
ModelNet40	IG vs. Grad-CAM	Correctness	<$10^{-64}$	0.597 (Large)
ModelNet40	Occlusion vs. Grad-CAM	Correctness	<$10^{-62}$	0.587 (Large)
ModelNet40	Saliency vs. Grad-CAM	Correctness	<$10^{-50}$	0.524 (Large)
ModelNet40	ResAttNet vs. IG	Correctness	<$10^{-58}$	0.469 (Medium)
ModelNet40	ResAttNet vs. Occlusion	Correctness	<$10^{-50}$	0.438 (Medium)
ModelNet40	ResAttNet vs. Saliency	Correctness	<$10^{-35}$	0.367 (Medium)

**Table 6:** Cross-domain quantitative synthesis of Correctness, Completeness, and Compactness metrics across datasets and explainability methods. Compactness is reported for both adaptive (a=0.1) and fixed (t=0.5) thresholds.

Dataset	Model	Method	Correctness	Completeness	Compactness (*a* = 0.1)	Compactness (*t* = 0.5)
MosMed	MedicalNet	Grad-CAM	0.5210	0.5110	0.9285	0.2371
MosMed	MedicalNet	Integrated Gradients	0.6887	0.5712	0.0742	0.0023
MosMed	MedicalNet	Occlusion	0.6615	0.6733	0.2554	0.0156
MosMed	MedicalNet	Saliency	0.6174	0.7769	0.0906	0.0012
MosMed	ResAttNet	Intrinsic	0.2365	0.5294	1.2907	0.9548
ScanObjectNN	PointNet++	Grad-CAM	0.6217	0.1012	0.1730	0.0974
ScanObjectNN	PointNet++	Integrated Gradients	0.6332	0.1028	0.0335	0.0011
ScanObjectNN	PointNet++	Occlusion	0.5928	0.1037	0.2184	0.0478
ScanObjectNN	PointNet++	Saliency	0.6275	0.1033	0.0561	0.0020
ModelNet40	VoxNet	Grad-CAM	0.4179	0.4733	(51.5919)	(21.7571)
ModelNet40	VoxNet	Integrated Gradients	0.7550	0.6843	0.4121	0.0511
ModelNet40	VoxNet	Occlusion	0.7377	0.8464	6.1019	0.4090
ModelNet40	VoxNet	Saliency	0.6865	0.7408	12.8060	0.2224
ModelNet40	ResAttNet	Intrinsic	0.3990	0.3743	(12.1165)	0.0002

## Data Availability

The data presented in this study are available: MosMedData (volumetric CT scans) is available in Kaggle Repository at https://www.kaggle.com/datasets/mathurinache/mosmeddata-chest-ct-scans-with-covid19 (accessed on 14 January 2026); ModelNet40 (voxelized CAD models) is available in Princeton ModelNet at https://modelnet.cs.princeton.edu/ (accessed on 14 January 2026); and ScanObjectNN (point-cloud objects) is available at https://hkust-vgd.github.io/scanobjectnn/ (accessed on 14 January 2026).
